# Editorial: Optical and electrochemical biosensing

**DOI:** 10.3389/fchem.2022.964825

**Published:** 2022-08-17

**Authors:** Cheng Ma, Tingting Zheng, Renyun Zhang, Jun-Jie Zhu

**Affiliations:** ^1^ School of Chemistry and Chemical Engineering, Yangzhou University, Yangzhou, Jiangsu, China; ^2^ School of Chemistry and Molecular Engineering, East China Normal University, Shanghai, China; ^3^ Department of Natural Sciences, Mid Sweden University, Sundsvall, Sweden; ^4^ State Key Laboratory of Analytical Chemistry for Life Science, School of Chemistry and Chemical Engineering, Nanjing University, Nanjing, Jiangsu, China

**Keywords:** biosensing, optical analysis, electrochemical analysis, photoelectrochemistry, microscopy

It is a great opportunity for us to organize the important Research Topic “*Optical and electrochemical biosensing*” that highlights the recent developments emerging in biosensing field. This Research Topic is here to help with a Research Topic of articles and reviews that cover recent optical and electrochemical biosensing technologies. Although many transduction approaches are available for biosensors, optical and electronic signals are still two important pillars of analysis. This Research Topic collects three articles on optical sensing and four articles on electrochemical sensing. In addition, some interconnection techniques between optical and electrochemical signals, such as photoelectrochemical sensor, demonstrated the merits of minimum intercross inference between input and output signals. The reviews are encouraged to cover the last 3 years developments so that readers can catch up on the most up to date trends in this Research Topic. Therefore, two reviews are included in the Research Topic to summary recent developments in analytical probes such as DNAzyme and metal graphitic nanocapsules. These reviews will also offer a convenient way for junior researchers to quickly understand a field.

Optical biosensing is one of the most important pillars in measurement science. By using light intensity, spectroscopy and microscopy, a fruitful source of information can be obtained from different aspects. The three articles on optical sensing in this Research Topic have chosen different fluorescent probes (gold nanocluster, organic molecules and quantum dots, respectively) for biosensing. The analytical targets range from neurotransmitters, ClO^−^, and glycoproteins on cell membranes. The most powerful information with optical output is its spatial resolution that enables the direct observation of the distribution of targets in cellular compartments. For example, the ferroptosis-induced ClO^−^ was mapped by using mitochondrial-targeted ratiometric fluorescent probe in confocal fluorescence microscope. Abundant groups in organic fluorescent molecules possess great flexibility in designing targeted units and reactions units. Thus, the recognition group p-methoxyphenol and mitochondrial-targeted group benzimidazole are integrated into the probe. Another fluorescence imaging strategy is to use fluorescence resonance energy transfer (FRET). A short distance between quantum dots and gold nanoparticles is controlled by the hybridization of aptamer and ssRNA. But antigen CD133 on the cell membrane competitively replaced ssRNA and bound to aptamer, releasing the gold nanoparticles and recovering the fluorescence signal. Thus, lung cancer cells can be determined by the FRET aptasensor using confocal imaging.

Electrochemical biosensing is another important issue in this Research Topic. Compared with fluorescence analysis, electrochemical techniques generally have a simple setup and higher sensitivity. The modified materials on electrode surface largely determined the performance of these electrochemical sensors. As we can see, the four collected articles about electrochemical sensors have emphasized the design of modified materials, including graphene, carbon nanotube, metal-organic frameworks. These materials not only fabricate conductive interface for electron transfer, but also act as vehicles for other nanomaterials such as noble metal nanoparticles. The combination of electrocatalytic materials and DNA amplification techniques further improves the sensitivity and limit of detection. For example, G-quatruplex-hemin hybridization chain reaction nanowire acted as a mimicking DNAzyme to assist the cycle amplification of electrochemical signals. Some biomarkers released by cells can also be detected by the nanomaterial-modified electrode.

In addition to individually used optical or electrochemical biosensors, interconnection techniques between optical and electrochemical signals demonstrate the unique advantages by separating input and output signals. Photoelectrochemical sensors attracted great attentions in recent years due to its low background. The semiconductor heterostructure plays a key role in the performance of photoelectrochemical signal sensitivity. Thus, novel bismuth-based semiconductors were used to fabricate photoelectrochemical interface for the detection of L-Cys. Compared with other analytical methods, photoelectrochemical analysis generally has a wide linear range and a low limit of detection. In addition to original articles, two reviews on DNAzyme and metal graphitic nanocapsules are included in this Research Topic. DNAzyme-based biosensor for the detection of uranyl is an important tool for monitoring the processes in nuclear technology. Diverse signal outputting strategies like fluorescence, colorimetry, surface-enhanced Raman scattering and electrochemistry are fully discussed in the realm of DNAzyme biosensing. In addition, the encapsulation of metal NPs is one of the most effective strategies to promote their stability for biosensing. Metal graphitic nanocapsules are excellent nanoprobes in many analytical methods. By using the properties of metal core and graphene shell, the Raman signal of graphene and catalytic activity of H_2_O_2_ in colorimetry analysis can be used in various biosensing.

I remember that every day writing at least five grateful things can increase the base level of happiness. Therefore, I would like to thank 1) our editorial team that informed the potential authors and put efforts on review processes, 2) the *Frontiers in Chemistry* staff that did excellent and professional works for publication in a timely manner, 3) the reviewers whose professional advice helped improve the quality of every publication, 4) the authors that contributed their efforts to develop new analytical techniques and summarize the recent progress, 5) our funding agencies that supported the financial cost in all experiments. It is both meaningful and pleasurable to complete this Research Topic and I appreciate if it is useful to our potential readers.

